# Subacromial Pain Syndrome in Breast Cancer Survivors—Are Structural Shoulder Changes Verified by Ultrasound Clinically Relevant?

**DOI:** 10.3390/diagnostics15010070

**Published:** 2024-12-30

**Authors:** Ivana Klarić-Kukuz, Jure Aljinović, Blaž Barun, Marko Roki, Benjamin Benzon, Danijela Budimir Mršić, Maja Marinović Guić, Ana Poljičanin

**Affiliations:** 1 Department of Physiotherapy, University Department of Health Studies, University of Split, 21000 Split, Croatia; ivanaklaric.k@gmail.com (I.K.-K.); ana.poljicanin@gmail.com (A.P.); 2 University Postgraduate Doctoral Study Program, School of Medicine, University of Split, 21000 Split, Croatia; blaz.barun@mefst.hr; 3 Department of Physical and Rehabilitation Medicine, School of Medicine, University of Split, 21000 Split, Croatia; 4 Institute of Physical Medicine and Rehabilitation with Rheumatology, University Hospital Split, 21000 Split, Croatia; marko.roki@hotmail.com; 5 Department of Anatomy, Histology and Embryology, School of Medicine, University of Split, 21000 Split, Croatia; benjamin.benzon@mefst.hr; 6 Department of Radiological Technology, University Department of Health Studies, University of Split, 21000 Split, Croatia; danijelabudimir@gmail.com (D.B.M.); maja.marinovic.guic@gmail.com (M.M.G.); 7 Department of Diagnostic and Interventional Radiology, School of Medicine, University of Split, 21000 Split, Croatia; 8 Department of Diagnostic and Interventional Radiology, University Hospital Split, 21000 Split, Croatia

**Keywords:** shoulder pain, rotator cuff, breast neoplasms, diagnostic imaging

## Abstract

**Background/Objectives**: Shoulder pain is a common treatment outcome in breast cancer survivors. While various risk factors and mechanisms for shoulder pain have been proposed, evidence is inconsistent. Increased risk of subacromial pain syndrome exists, which can lead to disability and reduced quality of life if untreated. Ultrasound is a valuable tool for detecting rotator cuff changes aiding in timely diagnosis of subacromial pain syndrome. This study aimed to assess the prevalence of rotator cuff changes to better understand chronic shoulder pain in breast cancer survivors. **Methods**: This cross-sectional study included 74 breast cancer survivors from the University Hospital Split. Data were collected via questionnaires and clinical interviews. Bilateral shoulder ultrasounds were performed by two blinded investigators. Categorical variables were analyzed using Chi-squared tests, and continuous variables were analyzed with T-tests or Mann–Whitney tests. **Results**: Pathological findings were similarly prevalent on the operated and non-operated sides (*p* = 0.3 and *p* = 0.6). Among participants with shoulder pain, ultrasound-detected pathology was present in 91% of right shoulders and 96% of left shoulders (*p* < 0.005). Non-painful shoulders exhibited pathology in 59% of right and 57% of left shoulders. Ipsilateral pain to the site of breast surgery was reported by 57.7% of participants, with supraspinatus pathology in 56%, acromioclavicular joint pathology in 39%, and subacromial–subdeltoid bursitis in 41%. **Conclusions**: Similar pathology distribution on operated and non-operated sides and frequent asymptomatic findings highlight unresolved causes of shoulder pain in breast cancer survivors. Ultrasound is valuable but requires integration with clinics for accurate diagnosis of the underlying causes of shoulder pain.

## 1. Introduction

According to cancer statistics published by the World Health Organization (WHO), breast cancer is the most prevalent cancer among women worldwide and accounts for the highest mortality rate among cancers [1,2]. However, advancements in targeted treatments have led to a steady increase in the number of breast cancer survivors (BCSs) [3]. In high-income countries, the current five-year survival rate for breast cancer has reached approximately 90% [3].

Despite the benefits of targeted breast cancer treatments, these therapies place survivors at risk of developing both short- and long-term structural and functional impairments. Such impairments can result in functional disability and a reduced quality of life [4,5]. Evidence suggests that nearly 90% of breast cancer survivors experience at least one impairment within six months post-treatment, and 62% continue to report impairments six years after completing treatment [6].

Given these findings, raising awareness about the challenges faced by survivors is essential to facilitate timely diagnosis, treatment, and rehabilitation. These considerations emphasize the critical need for multidisciplinary care across the cancer care continuum. This need has also been recognized by the WHO in its 2021 Global Breast Cancer Initiative, which advocates for comprehensive, patient-centered approaches to breast cancer management [7,8,9,10].

According to the International Consortium for Health Outcomes Measurement, upper limb dysfunction (ULD) is one of the most prominent treatment outcomes experienced by breast cancer survivors [11]. ULD significantly impacts survivors’ ability to perform activities of daily living, often resulting in a loss of independence, reduced capacity to work, and limitations in participating in social activities with family and friends. These challenges collectively contribute to a decreased overall quality of life [12,13,14,15].

Commonly reported ipsilateral ULDs vary widely in prevalence: shoulder/arm pain (up to 51%), limited shoulder range of motion (ROM) (up to 50%), decreased arm strength (25%), and lymphedema (6–52%), with prevalence rates depending on the treatment type and population studied [16,17,18,19,20,21]. Risk factors for ULD development include mastectomy, axillary lymph node dissection (ALND), radiation therapy, and higher body mass index (BMI) [22,23,24,25].

Chronic shoulder pain in breast cancer survivors is often attributed to various treatment-related factors [26]. Surgical procedures such as mastectomy and axillary lymph node dissection (ALND) can result in nerve damage, scar tissue formation, and altered shoulder mechanics, all of which contribute to persistent pain [27,28]. Radiation therapy may lead to fibrosis, restricting shoulder mobility and causing further discomfort [27]. Additionally, lymphedema, if present, can impair shoulder function and exacerbate pain [29]. These treatment-related factors often interact, complicating both diagnosis and management of chronic shoulder pain in breast cancer survivors. Therefore, it is crucial to identify the underlying cause of the pain in order to design an appropriate rehabilitation strategy.

Secondary to the above-mentioned treatment-related factors and ULDs, breast cancer survivors are considered at increased risk for developing subacromial pain syndrome, which encompasses various shoulder pathologies. Common conditions include supraspinatus tears (53.3%), biceps tenosynovitis (13.3%), and subdeltoid bursitis (13.3%) [27,30].

Timely diagnosis of subacromial pain syndrome (SAPS) in breast cancer survivors can be effectively facilitated using ultrasound. This imaging technique offers a non-invasive, cost-effective, and reliable method for assessing shoulder pathologies [31]. Ultrasound allows for real-time visualization of soft tissue structures, making it particularly valuable for diagnosing and monitoring the progression of SAPS [31]. Early detection through ultrasound is critical for guiding appropriate treatment and preventing long-term functional impairment in breast cancer survivors [31].

Although there is speculation in the scientific literature regarding a causative correlation between breast cancer treatment, shoulder pain, and subacromial pain syndrome, imaging studies addressing this topic are scarce, and their findings remain inconclusive [27,28,32,33,34]. Prevalence of symptomatic or asymptomatic rotator cuff pathology in breast cancer survivors is yet to be determined.

Notably, scarce small sample studies to date have systematically evaluated shoulder pathology and pain in breast cancer survivors [32,33,34].

To address this gap, the present imaging study was conducted to assess the prevalence of structural bilateral shoulder changes, with the aim of advancing the understanding of chronic shoulder pain in breast cancer survivors.

## 2. Materials and Methods

### 2.1. Study Population

**Figure 1:**
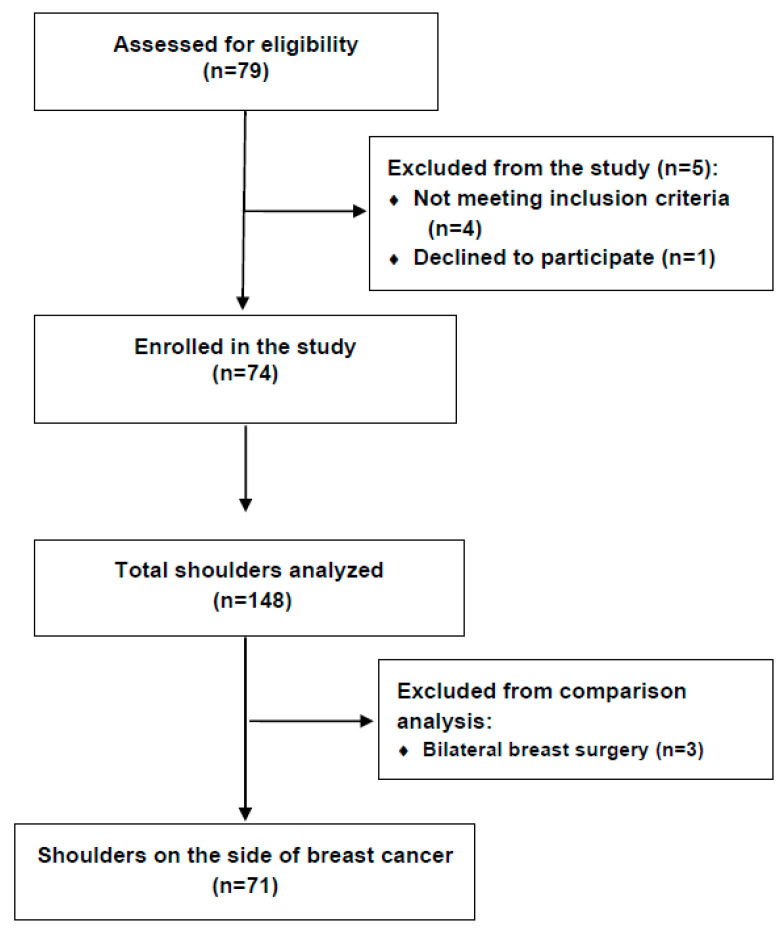
Flowchart of participants enrollment.

This cross-sectional observational study was conducted over a 6-month period from December 2023 to April 2024 on the Lymphedema Clinic breast cancer survivor’s cohort at the University Hospital Split, the second largest tertiary healthcare center in Croatia. This study included females aged 18 years and older who had completed treatment for unilateral breast cancer at least 6 months prior to enrollment and had consented to participate. Exclusion criteria included metastatic breast cancer, cognitive disorders, and a history of shoulder surgery. Following the application of exclusion criteria, a total of 74 breast cancer survivors were enrolled in this study (Figure 1).

This study was approved by the Ethical Committee of the University Hospital Split (protocol code 2181-147/01/06/LJ.Z.-23-2), and informed consent was obtained from all participants prior to enrolment.

### 2.2. Measurements and Data Collection

Baseline demographic information and comprehensive medical history were collected through self-administered questionnaires and clinical interviews. Missing data, if any, were supplemented by reviewing participants’ electronic medical records. Table 1 provides a summary of demographic and disease-related characteristics of the participants.

#### 2.2.1. Arm Circumference Measurement

Circumference measurements were performed by an experienced lymphedema therapist. Participants were seated with their arm resting on an adjustable hydraulic table, flexed at approximately 90° with the forearm pronated. Five measurements were taken at predetermined points starting at the ulnar styloid process of the wrist (designated as point 0), with additional measurements taken every 10 cm along the arm. Measurements were recorded in triplicate at each point using a flexible Juzo tape with 1 mm accuracy, and average values were documented on a standardized form. A patient was considered to have lymphedema if the circumference difference between the arms at any of five measured points was 2 cm or grater [35,36].

#### 2.2.2. Shoulder Ultrasonography

Shoulder ultrasonography was performed by two independent, experienced ultrasound specialists who were blinded to each participant’s current pain status and to each other’s assessments. Intraobserver variability was assessed using the intraclass correlation coefficient (ICC) for repeated measurements of 10% of shoulders, yielding an ICC of 0.9 (95% CI: 0.85–0.95), indicating excellent agreement. Scans were conducted using a 12 MHz linear probe (General Electrics HealthCare Versana Premier V2, Chicago, Illinois, USA) according to an established protocol [37]. All pathologies were scanned in two perpendicular probe positions. Both shoulders (n = 148) underwent ultrasound examination. The shoulders on the non-operated side served as controls. Three participants with bilateral breast surgery were excluded from the inter-shoulder comparison analysis, resulting in a final sample of 71 participants (Figure 1). The median interval between surgery and shoulder examination was five years (Table 1). The ultrasound images of shoulders were anonymized and independently reviewed, with any pathologies documented. For borderline findings, consensus was reached between the reviewers, and no further imaging was required. All measurements were taken from the coded, scanned images. Pathological findings in the rotator cuff were classified as tendinosis, calcific tendinopathy, and partial/full tears. Acromioclavicular joint (AC) arthrosis was graded from mild to severe. The presence of subacromial-subdeltoid bursitis (SASD), as well as effusion, tenosynovitis, or rupture in the long head of the biceps tendon (LHBT), was also recorded.

### 2.3. Statistical Analysis

Data were analyzed using Microsoft Office Excel 2016 32-bit (Microsoft Corporation, Redmond, WA, USA). Descriptive statistics were applied, with continuous data presented as medians with interquartile ranges (IQRs), and categorical data were presented as frequencies and percentages. Comparative analyses of categorical variables were conducted using the Chi-squared test, while differences in continuous variables were assessed with either the T-test or Mann–Whitney test, as appropriate. A significance level of α = 0.05 (*p* < 0.05) was used for all statistical tests.

## 3. Results

### 3.1. The Prevalence of Ultrasound Pathological Findings Identified in Shoulders of Breast Cancer Survivors

In the analysis of all shoulders (n = 148), the most frequently observed pathology was in the supraspinatus tendon (SSP), affecting 45% of all shoulders, followed by acromioclavicular (AC) joint arthrosis, observed in 35% of all shoulders. Pathological findings in the subscapularis (SSC), infraspinatus (ISP), and long head of the biceps tendon (LHBT) were present in 10–15% of all shoulders (Table 2).

The most common specific pathological finding was a tendon tear, with supraspinatus tendon tears identified in 37 shoulders (25%). Of these, 19 (12.8%) were partial tears, and 18 (12.2%) were full-thickness tears. Tears in other rotator cuff tendons were rare, observed in only five shoulders (3.4%) (Table 2).

The second most prevalent pathology was calcific tendinopathy, found in 28 shoulders (18.9%). Calcifications in multiple tendons were present in nine shoulders (6.1%). The SSP tendon was the most frequent site for calcifications (n = 18; 12.2%), followed by the ISP and SSC tendons, each affected in 10 shoulders (6.8%) (Table 2).

Adhesive capsulitis was diagnosed in 3 out of 74 patients, representing approximately 2% of the shoulders analyzed.

The prevalence of pathological ultrasound findings was comparable between right (n = 54, 73%) and left shoulders (n = 53, 72%) (Table 2). No statistically significant differences were identified in the type or frequency of pathologies between the right and left shoulders (*p*-values ranging from 0.4 to 0.76).

The only statistically significant difference between the right and left shoulders was the prevalence of SASD bursitis, which was present in 28.4% of right shoulders and 10% of left shoulders (*p* < 0.001) (Table 2).

The occurrence of SASD bursitis was not significantly associated with endocrine therapy (*p* = 0.427), chemotherapy (*p* = 0.721), or age (*p* = 0.344).

The prevalence of pathological findings was similar between operated and non-operated shoulders, for both the right and left sides (*p* = 0.3 and *p* = 0.6, respectively) (Table 2).

### 3.2. Prevalence and Ultrasound Characteristics of Painful Shoulders in Breast Cancer Survivors

At the time of ultrasound examination, 62% of participants reported shoulder pain, either on the operated or non-operated side. Further sub-analysis revealed that 18% reported pain in the left shoulder, 24% in the right shoulder, and 20% in both shoulders. Approximately one-third of patients (38%) reported no shoulder pain (Table 1).

Among patients reporting shoulder pain, ultrasound-detected pathology was present in approximately 90% of cases (91% for the right shoulder and 96% for the left shoulder). In contrast, non-painful shoulders exhibited pathology in 59% of right shoulders and 57% of left shoulders. A statistically significant correlation was confirmed between shoulder pain and pathological findings on ultrasound for both shoulders (*p* < 0.005).

Endocrine therapy was administered to 71.6% of patients, while anti-HER2 treatment was given to 16.2% (Table 1). Endocrine therapy was not associated with an increased prevalence of shoulder pain (*p* = 0.60). Similarly, breast cancer survivors who underwent radiotherapy (70%) and chemotherapy (56%) did not exhibit a higher prevalence of shoulder pain at the time of examination.

Shoulder pain was not significantly associated with body mass index (*p* = 0.636), chemotherapy (*p* = 0.47), radiotherapy (*p* = 0.571), endocrine therapy (*p* = 0.241), or dominant hand (*p* = 0.54).

### 3.3. Prevalence and Characteristics of Painful vs. Non-Painful Shoulder on the Side of Breast Surgery in Breast Cancer Survivors

Approximately 30% (n = 23) of participants reported experiencing early postoperative shoulder pain on the ipsilateral side of the breast surgery (Table 1). At the time of ultrasound examination, 57.7% (n = 41) participants reported pain in the shoulder on the side of breast surgery, while 42.2% (n = 30) reported no pain in the ipsilateral shoulder (Table 3). There was no significant difference between these groups concerning the time elapsed since surgery (*p* = 0.633).

In approximately half of the participants with ipsilateral shoulder pain, supraspinatus (SSP) pathology was identified in 56% of cases, followed by acromioclavicular (AC) joint pathology in 39% of cases, and subacromial–subdeltoid (SASD) bursitis in 41% of cases; other diagnoses accounted for the remainder of cases (Table 3).

The type of surgical procedure, whether radical mastectomy or breast-conserving surgery, as well as the extent of axillary intervention (radical axillary or sentinel lymph node dissection), did not significantly influence the prevalence of shoulder pain (*p* = 0.27).

Upper extremity lymphedema was observed in 35% of patients on the operated side (Table 1), yet it was not associated with an increased prevalence of shoulder pain compared to the contralateral side (*p* = 0.795).

## 4. Discussion

The underlying cause of shoulder pain in more than 90% of symptomatic cases is rotator cuff pathology; however, such pathology has also been identified in 50% of asymptomatic individuals. The most frequently observed lesion involves the supraspinatus (SSP) tendon, affecting 45% of examined shoulders, followed by acromioclavicular (AC) joint arthrosis, which was observed in 35% of shoulders. The most common specific pathological finding was tendon tear, with supraspinatus tendon tears identified in 37 shoulders (25%). Among these, 19 (12.8%) were partial-thickness tears, and 18 (12.2%) were full-thickness tears. Khoschnau et al. demonstrated that in the general population, only full-thickness rotator cuff tears should be prioritized for consideration, as these have a significant potential to impair shoulder function [38]. Studies have shown that the clinical presentation of rotator cuff tears exhibits significant variability and may present with or without associated symptoms [39,40,41]. Additionally, given that many tears are asymptomatic, there is a recognized risk of these lesions progressing to symptomatic states over time [42]. Consequently, ultrasound imaging plays a crucial role in the early diagnosis, monitoring, and planning of appropriate treatment strategies.

Pathological changes, such as acromioclavicular joint osteoarthritis and partial rotator cuff tears, are generally considered natural, age-related phenomena [38]. This may partially account for the observation that 50% of our patients were asymptomatic. Similar rotator cuff pathology was observed in both the operated and non-operated shoulders, suggesting an age-related etiology. As previously noted, these findings may be considered nonsignificant with respect to shoulder function [38].

Additionally, a substantial proportion of our patients (20%) exhibited bilateral pathology, consistent with findings from prior research on breast cancer patients [43,44,45]. Mafu et al. propose that the involvement of structures not directly affected by cancer treatment, combined with bilateral pathology, may indicate a systemic cause of the disease [46]. Hun Kim et al. suggest that bilateral pathology could also result from mechanical overload of the contralateral shoulder [28]. Similarly, rotator cuff pathology was equally present in both operated and non-operated shoulders. Which could be age related and, as previously stated, are nonsignificant findings considering shoulder function [38]. These findings underscore the importance of regular bilateral shoulder ultrasound examinations for early detection and comprehensive assessment.

In our study, we did not identify any correlation between known risk factors, such as cancer treatment, body mass index (BMI), hand dominance, or lymphedema, and the development of shoulder pain. This finding aligns with previous research by Hun Kim and Yang et al. (2010) on rotator cuff-related shoulder pain [32,33,34]. Study design limitations, including a small sample size and limited sample diversity, have been proposed as potential explanations for these results [32]. Furthermore, it is important to recognize that shoulder pain and associated pathologies may not result solely from cancer treatment itself but rather from its long-term consequences. These may include pectoralis muscle tightness, fibrosis, neuropathic changes, and misalignment of the shoulder girdle, which collectively contribute to altered biomechanics and pain [27].

Although ultrasound imaging has been shown to be a valuable diagnostic tool, it should be taken into account that subacromial pain syndrome is a multifactorial condition, and reliance solely on pathological findings in the rotator cuff is insufficient for a proper diagnosis. Other physical and psychological factors may significantly influence the severity and presentation of the disease, underscoring the importance of a comprehensive clinical evaluation [47].

### Study Limitations

Our study has several limitations that warrant consideration:

Sample size and control group: the study sample was relatively small, and the control group could be expanded to include healthy women matched by age. This limitation restricts the generalizability of the findings and highlights the need for a larger, more representative cohort in future studies.

Cross-sectional design: as a cross-sectional study, we were unable to determine the temporal relationship between exposure and outcomes since data were collected at a single time point. This limits our ability to establish causality. Future research should adopt longitudinal designs with extended follow-ups from diagnosis to at least 2–5 years post-treatment to assess causality and long-term outcomes of painful shoulder conditions and rotator cuff pathology.

Despite efforts to maintain blinding during ultrasonography examination, the physical interaction required during ultrasonography could introduce detection bias.

Characterization of subacromial pain syndrome: in this study, subacromial pain syndrome was defined primarily by pain, without addressing other critical components such as limited range of motion, muscle weakness, functional impairments, and quality of life. Inclusion of these factors in future studies would provide a more comprehensive understanding of upper limb dysfunction.

Extrinsic factor analysis: detailed analysis of extrinsic factors such as shoulder girdle alignment, altered kinematics, and muscle performance was not included in our study. Future research should explore these aspects to better understand their impact on shoulder pathology and rehabilitation outcomes.

Addressing these limitations in future studies will enhance the robustness and clinical applicability of findings in this domain.

## 5. Conclusions

This study demonstrates a high prevalence of rotator cuff pathology in breast cancer survivors, regardless of the breast surgery side. The most commonly observed lesions involved the supraspinatus tendon, followed by acromioclavicular joint arthrosis, with significant pathology detected in both symptomatic and asymptomatic individuals. The similar distribution of pathology on both sides suggests that age-related changes may contribute to these findings, independent of cancer treatment.

The absence of a clear correlation between shoulder pain and known risk factors such as cancer treatment, BMI, hand dominance, or lymphedema highlights the complexity of chronic shoulder pain in breast cancer survivors. These findings emphasize that shoulder dysfunction likely arises from multifactorial causes, including long-term treatment effects, altered biomechanics, and systemic or mechanical factors.

While ultrasound remains a valuable tool for detecting structural changes, its findings must be interpreted within a comprehensive clinical context, as subacromial pain syndrome is a multifactorial condition. A multidisciplinary approach that integrates imaging, physical examination, and consideration of psychological factors is essential for accurate diagnosis and effective management of shoulder pain in breast cancer survivors.

Future research with larger and more diverse cohorts is needed to further clarify the underlying mechanisms of shoulder pain in breast cancer survivors and to optimize treatment strategies aimed at improving functional outcomes and quality of life in this population.

## Figures and Tables

**Table 1:** Participants’ demographics and disease-related characteristics.

Participants	*n*	%
Age (Median, IQR)	57 (50–64)	100
BMI ^1^		
18.5–24.9 kg/m^2^	25	33.78
25–29.9 kg/m^2^	31	41.89
≥30 kg/m^2^	18	24.32
Age at BC ^2^ diagnosis (Median, IQR)	52 (45–57)	100
Time since BC ^2^ surgery (Median, IQR)	5 (2–9)	100
Operated side:		
Right	37	50
Left	34	45.95
Both	3	4.05
Type of surgery:		
Mastectomy	47	63.51
Breast-conserving	27	36.49
Breast reconstruction:		
Yes	20	27.02
No	54	72.97
Lymph node removal:		
SLND ^3^	27	36.49
ALND ^4^	46	62.16
Operation on a dominant side:		
Yes	46	62.16
No	28	37.84
Early postoperative ipsilateral shoulder pain:		
Yes	23	31.08
No	49	66.22
After-surgery complications:		
Yes	54	72.97
No	18	24.32
Radiotherapy:		
Yes	52	70.27
No	22	29.73
Chemotherapy:		
Yes	42	56.80
No	32	43.20
Anti HER2 treatment	12	16.22
Endocrine treatment	53	71.62
Any shoulder pain at present:		
Yes	46	62.16
No	28	37.84
Self-report of hand swelling:		
Yes	40	54.05
No	34	45.94
Established diagnosis of lymphedema (cm):		
Yes	27	35.06
No	57	74.02

^1^ Body mass index, ^2^ breast cancer, ^3^ sentinel lymph node dissection, and ^4^ axillary lymph node dissection.

**Table 2:** Bilateral ultrasound pathological findings in shoulders of breast cancer survivors.

		Total (n = 74)				Operated vs. Control Side (n = 71)				Operated vs. Control Side (n = 71)			
Structure	Pathology type	L ^1^		R ^2^		L ^1^-Operated		L ^1^-Control		R ^2^-Operated		R ^2^-Control	
		n = 74	%	n = 74	%	n = 33	%	n = 38	%	n = 38	%	n = 33	%
AC ^3^ joint	no pathology	52	70.3	45	60.8	20	60.6	29	76.3	25	65.8	17	51.5
	mild arthrosis	12	16.2	15	20.3	8	24.2	4	10.5	7	18.4	8	24.2
	≥moderate arthrosis	10	13.5	14	18.9	5	15.2	5	13.2	6	15.8	8	24.2
LHBT ^4^	no pathology	64	86.5	65	87.8	31	93.9	31	81.6	34	89.5	28	84.8
	effusion	7	9.5	6	8.1	2	6.1	4	10.5	1	2.6	5	15.2
	tenosynovitis	2	2.7	3	4.1	0	0.0	2	5.3	3	7.9	0	0.0
	rupture	1	1.4	0	0.0	0	0.0	1	2.6	0	0.0	0	0.0
SSC ^5^	no pathology	65	87.8	61	82.4	29	87.9	33	86.8	33	86.8	25	75.8
	partial tear	1	1.4	1	1.4	0	0.0	1	2.6	1	2.6	0	0.0
	full tear	0	0.0	0	0.0	0	0.0	0	0.0	0	0.0	0	0.0
	calcification	4	5.4	6	8.1	2	6.1	2	5.3	2	5.3	4	12.1
	tendinosis	4	5.4	6	8.1	2	6.1	2	5.3	2	5.3	4	12.1
SSP ^6^	no pathology	41	55.4	40	54.1	19	57.6	21	55.3	18	47.4	20	60.6
	partial tear	7	9.5	12	16.2	1	3.0	4	10.5	9	23.7	2	6.1
	full tear	10	13.5	8	10.8	5	15.2	5	13.2	5	13.2	3	9.1
	calcification	9	12.2	9	12.2	4	12.1	5	13.2	5	13.2	4	12.1
	tendinosis	7	9.5	5	6.8	4	12.1	3	7.9	1	2.6	4	12.1
ISP ^7^	no pathology	65	87.8	66	89.2	28	84.8	34	89.5	34	89.5	29	87.9
	partial tear	0	0.0	0	0.0	0	0.0	0	0.0	0	0.0	0	0.0
	full tear	1	1.4	1	1.4	0	0.0	1	2.6	1	2.6	0	0.0
	calcification	5	6.8	5	6.8	4	12.1	1	2.6	3	7.9	2	6.1
	tendinosis	3	4.1	2	2.7	1	3.0	2	5.3	0	0.0	2	6.1
SASD ^8^ bursitis	no	66	89.2	53	71.6	28	84.8	35	92.1	22	57.9	28	84.8
	yes	8	10.8	21	28.4	5	15.2	3	7.9	16	42.1	5	15.2

^1^ left (L), ^2^ right (R), ^3^ acromioclavicular joint (AC), ^4^ long head of the biceps tendon (LHBT), ^5^ subscapularis tendon (SSC), ^6^ supraspinatus tendon (SSP), ^7^ infraspinatus tendon (ISP), and ^8^ subacromial-subdeltoid bursitis (SASD).

**Table 3:** Comparison of ultrasound findings in painful vs. non-painful shoulders on the side of breast surgery in breast cancer survivors.

		Painful (n = 41)		Non-Painful (n = 30)	
		n	%	n	%
AC joint	no pathology	25	61%	20	67%
	pathology	16	39%	10	33%
	mild arthrosis	11	27%	4	13%
	moderate/severe arthrosis	5	12%	6	20%
LHBT	no pathology	37	90%	28	94%
	pathology	4	10%	2	6%
	effusion	2	5%	1	3%
	tenosynovitis	2	5%	1	3%
Subscapularis	no pathology	33	80%	29	97%
	pathology	8	20%	1	3%
	partial tear	1	2%	0	0%
	calcific tendinopathy	4	10%	0	0%
	tendinosis	3	7%	1	3%
Supraspinatus	no pathology	14	34%	23	77%
	pathology	27	56%	7	23%
	partial tear	9	22%	1	3%
	full tear	8	20%	2	7%
	calcific tendinopathy	6	15%	3	10%
	tendinosis	4	10%	1	3%
Infraspinatus	no pathology	36	88%	26	87%
	pathology	5	12%	4	13%
	full tear	1	2%	0	0%
	calcific tendinopathy	3	7%	4	11%
	tendinosis	1	2%	0	0%
SASD bursitis	no	24	59%	26	87%
	yes	17	41%	4	13%
Shoulders with calcification	no	33	80%	23	77%
	yes	8	20%	7	23%

## Data Availability

The datasets presented in this article are not readily available because they are part of an ongoing study.
